# Synchronous Generative Development amidst Situated Entropy

**DOI:** 10.3390/e24010089

**Published:** 2022-01-05

**Authors:** Stephen Fox

**Affiliations:** VTT Technical Research Centre of Finland, FI-02150 Espoo, Finland; stephen.fox@vtt.fi; Tel.: +358-40-747-8801

**Keywords:** active inference, agroecology, federated digital twins, free energy principle, generative model, generative process, joint agent-environment systems, situated entropy, synchronicity

## Abstract

The Sustainable Development Goals have been criticized for not providing sufficient balance between human well-being and environmental well-being. By contrast, joint agent-environment systems theory is focused on reciprocal synchronous generative development. The purpose of this paper is to extend this theory towards practical application in sustainable development projects. This purpose is fulfilled through three interrelated contributions. First, a practitioner description of the theory is provided. Then, the theory is extended through reference to research concerned with multilevel pragmatics, competing signals, commitment processes, technological mediation, and psychomotor functioning. In addition, the theory is related to human-driven biosocial-technical innovation through the example of digital twins for agroecological urban farming. Digital twins being digital models that mirror physical processes; that are connected to physical processes through, for example, sensors and actuators; and which carry out analyses of physical processes in order to improve their performance. Together, these contributions extend extant theory towards application for synchronous generative development that balances human well-being and environmental well-being. However, the practical examples in the paper indicate that counterproductive complexity can arise from situated entropy amidst biosocial-technical innovations: even when those innovations are compatible with synchronous generative development.

## 1. Introduction

Situated entropy encompasses thermodynamic entropy, statistical physics entropy, and information-theoretic entropy. The thermodynamic entropy of energy being unavailable to perform productive work is situated in different experiences of physical disorder (statistical physics entropy) and information uncertainty (information-theoretic entropy). Consider, for example, the different experiences of situated entropy that can arise from replacement of tropical forest with areas of industrial agriculture. Initially, the tropical forest experiences [1,2] extreme physical disorder and information uncertainty when its trees are ripped up. Hence, situated entropy is high for the forest. Conversely, people engage in industrial agriculture experience low physical disorder and low information uncertainty when using chemicals on the newly constructed flat fields and when driving lorries on the newly constructed hard paved roadways. Hence, situated entropy is initially low for people. However, over time, chemical fertilizers and pesticides can undermine both soil health and human health, which results in increased physical disorder and information uncertainty for both [3,4]. At the same time, the underside of new roadways can soon crack because of ground heave due to heavy rains, and the forest can begin to grow back through the road. This can lead to people experiencing increased physical disorder and information uncertainty as they have to drive around wide cracks in the road. As the road deteriorates and the forest tries to regrow through the road cracks, there can be more balance between situated entropy experienced by people and situated entropy experienced by the environment compared to when the road was first built. Yet, it is not a positive balance because it is a balance in which neither thrives. Indeed, transportation problems are a major cause of post-harvest losses [5,6]. More energy can be expended to try to minimize peoples’ situated entropy. For example, more energy could be expended in repeated road repairs. Alternatively, even more energy could be expended by ripping up the entire forest to stop it impinging on human activities. However, such imbalance between very low situated entropy experienced by people and very high situated entropy experienced by environments is also not positive. This is because systems characterized by very low entropy can be vulnerable to disturbances [7,8], and disturbances are inevitable [9,10], including natural disturbances that can destroy well-constructed infrastructure in a few hours [11].

Apropos, although most Sustainable Development Goals (SDGs) are focused on human development, they do include some consideration of environmental well-being. For example, there are goals for “Life Below Water” and for “Life On Land”. The SDGs are 17 interlinked global goals, which were set up in 2015 by the United Nations General Assembly and are intended to be achieved by the year 2030. However, there has been criticism that the SDGs do not provide sufficient balance between human well-being and environmental well-being. For example, it has been argued that SDGs do not address conflict between economic growth and environmental sustainability [12,13]. By contrast, joint agent-environment systems theory is focused on reciprocal synchronous generative development [14,15]. The purpose of this paper is to extend this theory towards practical application in sustainable development projects: in particular, those involving digital twins [16,17].

This purpose is fulfilled through three contributions. The first is providing a description for practitioners. This is necessary because extant descriptions of joint agent-environment systems rely upon specialist terminology of neuroscience and theoretical biology [14,15]. Hence, they are of limited usefulness for the broad range of people who may contribute to sustainable development projects involving digital twins. Second, main constructs in joint agent-environment systems are extended. This is necessary because extant descriptions of joint agent-environment systems do not encompass the complexity of human-driven biosocial-technical systems. Rather, they deal with simpler cases such as earthworms changing the soil they inhabit [15]. Third, joint agent-environment theory is related to federated digital twins. That is sets of digital models that mirror physical processes; that are connected to physical processes, for example, through sensors and actuators; and which carry out analyses of physical processes in order to improve their performance. Federated digital twins comprise several different digital twins that share data [16,17]. The federated digital twins example is of different types of agroecological urban farming [18]. Agroecology is a term used to describe human agricultural systems that take into account interactions between plants, animals, people, and the environment. For example, agroecology encompasses the well-being of soil and people in the cultivation and processing of food in and around urban areas [19]. Agroecology integrates scientific discipline, social movement, and sustainable practice, all of which are relevant to SDGs [20]. Moreover, agroecology is very relevant to joint agent-environment systems as it involves the coupling of human and natural processes [21].

Joint agent-environment systems theory is extended towards practical application in sustainable development projects through the contributions in the remaining five sections of this paper. Next, in Section 2, an overview of previous studies relating entropy to sustainable development is provided. In addition, the potential progress beyond the state-of-the-art provided by joint agent-environment systems theory is discussed. Then, in Section 3, a description of joint agent-environment systems theory is provided for development project practitioners. Subsequently, in Section 4, theoretical extensions are explained. In Section 5, the extended theory is related to federated digital twins for agroecological urban farming. In conclusion, principal contributions are stated and directions for future research are discussed in Section 6.

## 2. Entropy Studies and Sustainable Development

The 2015 Sustainable Development Goals (SDGs) are the latest formalization of global statements about the need for sustainable development. They follow from the Millennium Development Goals (MDGs), which were eight international development goals that were established following the Millennium Summit of the United Nations in 2000. Only one of the eight MDGs addressed environmental sustainability, and the MDGs did not address conflict between economic growth and environmental sustainability [22]. The MDGs lack of emphasis on environmental sustainability came 23 years after the World Commission on Environment and Development report Our Common Future was published by the United Nations through Oxford University Press [23]. This followed the 1972 bestselling book The Limits to Growth: A Report for the Club of Rome’s Project on the Predicament of Mankind [24]. In The Limits to Growth, its authors concluded that if humanity kept pursuing economic growth without regard for environmental costs, global society would experience a sharp decline in economic, social, and environmental conditions. Subsequent analyses support the book’s forecasts [25]. Amidst decades of global statements about the need for sustainable development, agroecology has emerged as a conceptualization of agriculture based on intrinsic coupling of human and natural processes [21].

Over the decades of global statements about the need for sustainable development, there have been many related entropy descriptions. One has been described as entropy pessimism. This description is based on thermodynamic entropy, within which all matter and energy is transformed from states available for human purposes, such as natural resources, to states unavailable for human purposes, such as pollution and waste [26,27]. Apropos, it has been argued that thermodynamic entropy excess is a “fee” that has to be paid by society for the use of modern industrial technologies in agriculture [28], and that the rate of thermodynamic entropy production is an important measure in assessing sustainability [29]. At the same time, information-theoretic entropy has been applied to describe different aspects of sustainability including power systems [30], urbanization [31], and water resources [32]. The entropy weight coefficient method has been applied. Within this information-theoretic method, the smaller the entropy value, the greater the weight of a performance indicator. The method has been applied in the formulation of SDG-based indices to calculate the weights of development performance indicators [33]. More broadly, information-theoretic entropy has been included in analyses of sustainability that focus on the need for there to be flexibility as well as efficiency in ecosystems [7]. Thermodynamic entropy [34] and information-theoretic entropy [35] have been applied separately in the description of human-nature coupled systems. However, the most comprehensive account is provided by joint agent-environment systems theory with its focus on reciprocal synchronous generative development [14,15] and its foundation in physics of life principles [36,37], which have been applied in analyses of human [38,39] and natural systems [40,41]. Addressing entropy is at the core of these formulations. In particular, addressing information-theoretic entropy can enable biological systems to maintain their sensory states within sustainable physiological states and so resist the second law of thermodynamics by which entropy tends towards a maximum.

Within the physics of life formalisms that underlie joint agent-environment systems [14,15], it is postulated that the brain embodies an internal model of the world that is generative in that it can simulate the sensory data that it should receive if its model of the world is correct. These simulated (i.e., predicted) sensory data can be compared to actual observations, and differences between predicted and observed sensations can be used to update the model [42]. Similarly, analyses carried out by digital twins often involve model-based simulations [43]. Moreover, key constructs in agent-environment systems’ generative models are important for digital twin simulation models. In particular, there is a trade-off between accuracy and complexity, within which the model should be as accurate as possible, but complexity must be minimized in order to facilitate reliable economic updating [44]. The model should also be focused on minimizing risk to the operation of the physical processes and on minimizing the ambiguity of data collected from the physical processes. Minimizing risk and ambiguity being necessary to facilitate survival in a changing world [45]. Thus, joint agent-environment systems theory provides a first principles account of coupling of human and natural processes, which is relevant to digital twins. Hence, joint agent-environment systems theory is the focus of this paper.

## 3. Synchronous Generative Agent-Environment Systems

In this section, a description is provided for practitioners. This is necessary because extant descriptions of joint agent-environment systems rely upon specialist terminology and mathematics of neuroscience and theoretical biology [14,15]. Hence, they are of limited usefulness for the broad range of people who may contribute to sustainable development projects involving digital twins. These can include local people with different backgrounds who have valuable knowledge and skills in agroecological practice but have low levels of functional and digital literacy due to divides in education and technology access. Participants can also include non-government organizations’ (NGOs) project staff and academic institutions’ computer scientists who can be equally unfamiliar with specialist terminology and mathematics of neuroscience and theoretical biology. Accordingly, descriptions involving widely used visual formats, such as radar charts, can be useful to provide a basis for working towards a shared understanding of joint agent-environment systems. An example of this would be when local people, project staff, and computer scientists need to have a shared understanding from which to begin the co-design of urban agroecology, digital twins, and interactions between them. Thus, visual formats are used in this section. First, description is provided of varying synchronization between agents and environments. Then, there is description of interactions between generative models and generative processes.

### 3.1. Varying Synchronization

**Figure 1:**
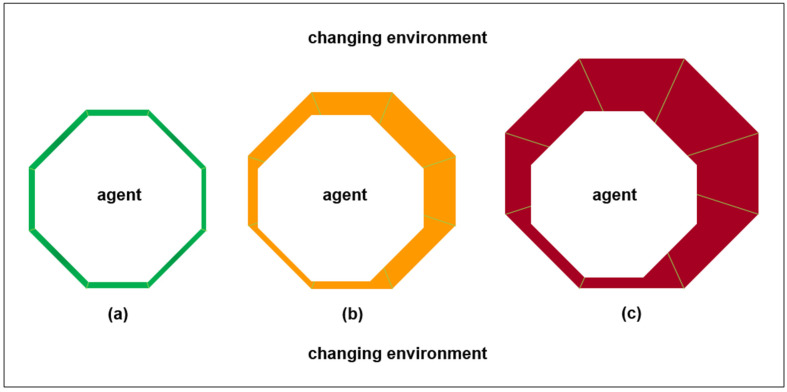
Decreasing agent-environment synchronization. (**a**) High synchronization: agent and environment share attracting synchronization manifold, which entails low survival information deficit at the interfaces between the agent and the environment (green). (**b**) Medium synchronization: agent does not change but environment changes, which entails medium survival information deficit at the interfaces between agent and environment (orange). (**c**) Low synchronization: agent does not change but environment changes increase, which entails high survival information deficit at the interfaces between the agent and the environment (red), so the agent cannot survive unless the agent improves its fit with environment, or the agent migrates to another environment.

Environments consist of multiple agents that mutually constrain each other until an attracting synchronization manifold is reached [15]. An attracting manifold is a set of states toward which a system tends to evolve from a wide variety of starting conditions. Synchronization among agents in an attracting manifold involves them remaining close to each other even when there are disturbances. Figure 1 illustrates varying agent-environment synchronization. In Figure 1a, there is high synchronization as there is a good fit between the agent and the environment. There can be little physical disorder and little information uncertainty for the agent and for the environment in such a situation. Hence, unproductive energy expenditure can be low, and there can be ample energy available to perform productive work. In Figure 1b, there is medium synchronization because the agent does not change but the environment changes. In Figure 1c, there is low synchronization because the agent still does not change, but environment changes increase. In such a situation, there can be very high situated entropy for the agent and for the environment because both experience high physical disorder and high information uncertainty. Hence, unproductive energy expenditure can be high and there can be little energy available to perform productive work. Hence, the agent cannot survive unless the agent improves its fit with the environment, or the agent migrates to another environment.

Figure 1 illustrates that synchronization is manifold because synchronization involves multiple survival parameters (i.e., multiple fitness components [46]). In Figure 1, there are eight survival parameters. For example, the survival parameters of a person can be for physiology, safety, social belonging, esteem, creativity, aesthetics, self-actualization, and transcendence [47]. As such parameters have different quantitative metrics and different qualitative descriptions, varying survival information deficit (i.e., variational free energy [39]) cannot be calculated conclusively. Moreover, it cannot be calculated conclusively because the agent cannot have perfect knowledge of the environment. Rather, many details of the environment can be described as being hidden [15].

**Figure 2:**
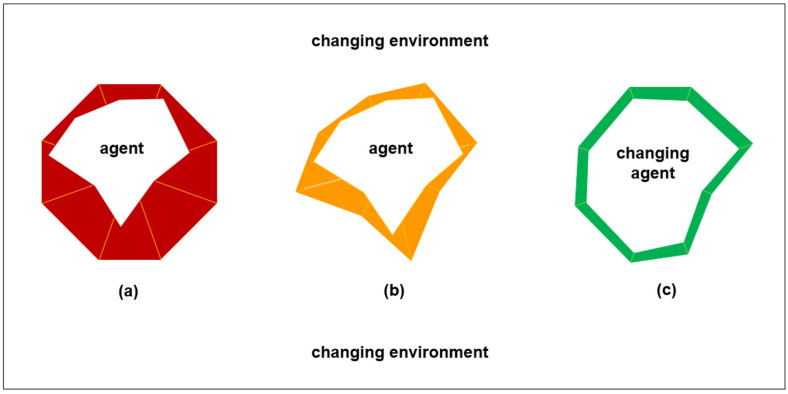
Increasing agent-environment synchronization. (**a**) Low synchronization: agent and environment have low synchronization, which entails high survival information deficit at interfaces between agent and environment (red). (**b**) Medium synchronization: the agent does not change itself, but the agent changes the environment, which entails medium survival information deficit at interfaces between agent and environment (orange). (**c**) High synchronization: the agent changes and the environment changes, which entails minimal survival information deficit at interfaces between agent and environment (green).

Figure 1 illustrates deteriorating agent-environment synchronization. However, as illustrated in Figure 2 below, agents and environments can learn and develop together. For example, forest gardens are highly resilient agroecosystems that involve a gradual process of people improving their immediate environment through the protection of useful bush, palm, tree, and vine species. These agroecosystems have been productive since prehistoric times [48]. In such situations, agent and environment can be equally flexible in learning about each other and developing together. Figure 2a illustrates that an agent initially has a high survival information deficit when it arrives in a new environment and hence has low synchronization. Figure 2b illustrates that the agent tries to change the environment in order to reduce the ambiguity of sensory inputs from the environment. This is done to increase the expected accuracy of sensory inputs from the environment. For example, expected accuracy of food yields from different species of bushes, palms, trees, and vines. This can involve reducing some species that yield food more erratically than other species. Figure 2b illustrates that changing the environment reduces the agent’s survival information deficit, but there is still not high synchronization.

Figure 2c illustrates that the agent learns more about the environment and changes itself in order to improve synchronization with the environment to reduce the risk of not surviving. For example, the agent can expand the range of bushes, palms, trees, and vines that it will eat from if that is the only way to survive. This can reduce the complexity of survival if it leads to simple nearby provision of sufficient food. Such varying of survival information deficit (i.e., variational free energy [39]) can be thought of as a varying trade-off between complexity and accuracy to achieve futures that are minimally risky and minimally ambiguous. As illustrated by Figure 2c, the agent changing itself can involve the agent changing its boundaries with the environment. This involves changing the scope of its internal states and the actions that it seeks to have autonomy over [36,37]. Changing of boundaries can balance transaction costs between work done internally by the agent and work done externally in the environment [49].

### 3.2. Generative Models and Generative Processes

**Figure 3:**
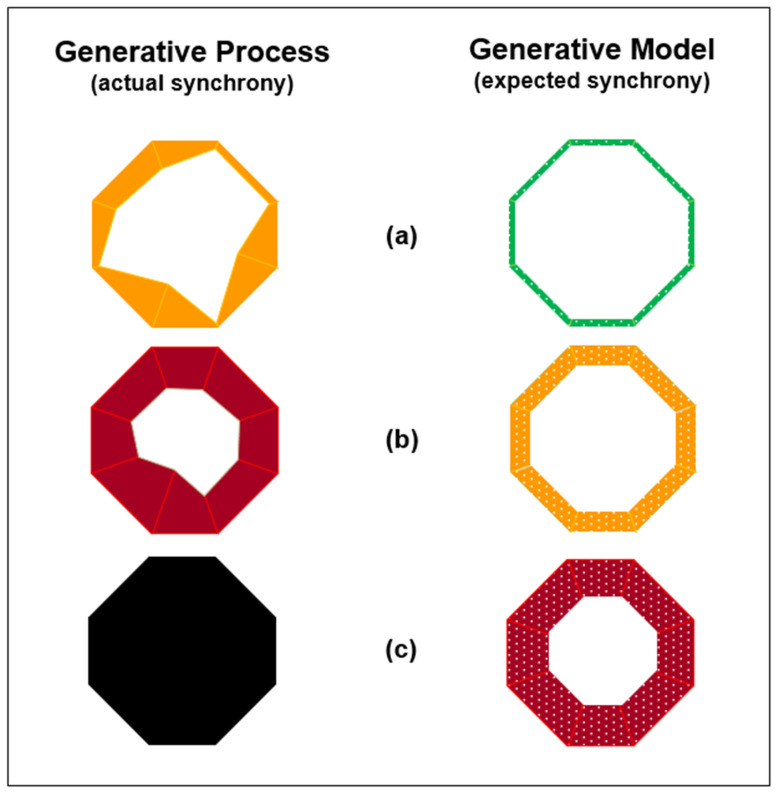
Expected synchrony versus actual synchrony. (**a**) Expected survival information deficit in generative model is minimal but actual survival information deficit in generative process is medium. (**b**) Agent updates expected survival information deficit to medium, but the actual survival information deficit is high. (**c**) Agent updates expected survival information deficit to high, but the actual survival information deficit is too high for the agent to survive.

As illustrated in Figure 3, this mutualistic flexibility can be represented as reciprocal oscillating interactions between agents’ generative models of themselves in the world and the generative process of themselves in the actual world. Agents’ generative models generate their expectations about the world, while the generative process generates agents’ observations of the world [15,40,41]. However, in other situations, one agent may be less flexible than others. In industrial agriculture, for example, people are the driver of joint dynamics. Initially, this can benefit people at the expense of the environment. However, through circular causation, it can come to be at the expense of people as well. For example, people engaged in industrial agriculture experience low physical disorder and low information uncertainty when using chemicals on newly constructed flat fields. Hence, situated entropy is initially low for people. However, over time, chemical fertilizers and pesticides can undermine both soil health and human health, which results in increased physical disorder and information uncertainty for both [3,4,50,51].

Nonetheless, as illustrated in Figure 3, some agents will persist with a course of actions (i.e., a policy) even when its unsustainable consequences have been apparent for a long time [52]. For example, agents can be committed to overgrazing [53,54]. Figure 3a illustrates the actual survival information deficit of an agent being worse than the minimal survival information deficit the agent had expected. Figure 3b illustrates the agent’s actual survival information deficit being more than the agent’s updated expectation for tolerable survival information deficit. Figure 3c illustrates that the agent does not survive because, although the agent updated its expectations, it did not update its policies about how to survive. Rather, instead of changing the structure of its generative model by introducing new survival parameters and new policies, the agent persisted with the same policies on the same survival parameters. As well as the agent not surviving, the environment may not survive because of the agent’s persistence [55]. Overall, Figure 3 illustrates that there will not be high synchronicity when a generative model does not resemble a generative process [15].

The more commitment an agent has to a policy, the more stubborn an agent can be in persisting with actions based on the same old policy irrespective of the agent experiencing observations that indicate continuing with the policy is counterproductive. Within joint agent-environment systems theory, the technical term concentration parameter is used [15] rather than the everyday word commitment. Paradoxically, commitment to a failing course of action can be consistent with there being a varying trade-off between complexity and accuracy in order to achieve futures that are minimally risky and minimally ambiguous. This can happen if an agent seeks to reduce complexity by paying more attention to its familiar policy than to observations that indicate continuing with the policy is counterproductive. Here, it is important to note that human identities can be symbolized by specific livestock and/or specific crops, which are strongly associated with their survival histories and their cultural values [56,57]. Hence, an agent can focus on reducing the existential risk to identity by ignoring observations that indicate continuing with the policy is counterproductive. Thus, the trade-off between complexity and accuracy involves focusing on the risky reward of continuing with the policy from which identity is derived and ignoring ambiguity arising from the expected inaccuracy of sensory inputs from the environment.

Agents can be considered to be dynamical living systems that survive through maintaining a near-to equilibrium steady state in relation to environmental fluctuations. Within this framing, continuing to pay more attention to a familiar policy than to observations indicating that the policy is counterproductive can be considered in terms of conserving flows and dissipative flows that exchange energy and matter with the external environment. For example, soil-plant systems exchange both energy and matter with their surroundings and are consequently open systems that tend towards a non-equilibrium steady state that is characterized by minimum production of entropy [36,37,58,59]. Where there are small amplitude environmental fluctuations, conserving flows cause the agent to revisit the same regions repeatedly through what can be described as homeostatic processes. By contrast, large amplitude fluctuations need to be counteracted by dissipative flows that enable the living system to establish a new near-to equilibrium steady state through what can be described as allostatic processes. Allostasis is the process of achieving internal stability through physiological or behavioral change—in contrast with homeostasis, which maintains internal stability by maintaining the organism’s internal state at a set point [60]. Persisting with homeostatic conserving flows amidst large amplitude fluctuations can lead to the agent being in an unsustainable stable steady state.

In this context, Figure 3c illustrates what can be described as oscillation death. This involves the end of back-and-forth reciprocal exchanges of agent-environment learning and development, in which synchronous near-to equilibrium steady-states are sustained by both. Instead, oscillation death involves agent and environment forming stable steady-states that undermine the survival of both. For example, neither agent nor environment may be able to survive overgrazing. Moreover, overgrazing can decouple agent and environment, leading to the loss of general oscillation synchrony between them. This can be described in terms of rhythmogenesis. That is the generation of rhythms found in most of the coupled physical, chemical, and biological systems in which underlying coupling acts as a feedback factor [61]. For example, sustainable agricultural practices need to involve people synchronizing with the environment’s seasonal rhythms [62].

A summary of important constructs is provided in Table 1. These are generative process, generative model, observations, commitment, actions, accuracy, complexity, risk, ambiguity, and synchrony.

## 4. Theory Extension

Extant descriptions of joint agent-environment systems do not encompass the complexity of observations, commitment, and actions in human-driven biosocial-technical systems. Rather, they provide illustrative examples such as earthworms changing the structure and chemical composition of the soil they inhabit [15]. Accordingly, in this section, the construct of observations is extended in terms of multilevel pragmatics [63] and competing signals [64]. Next, the construct of concentration parameters is extended in terms of commitment processes [65]. Then, the construct of actions is extended in terms of technological mediation [66] and psychomotor functioning [67]. Subsequently, a synthesis is provided.

### 4.1. Observations: Multilevel Pragmatics and Competing Signals

Within joint agent-environment systems theory, the observations of an agent are caused by the generative process of the environment. Pragmatics is concerned with how context influences the meanings of observations. Pragmatics encompass three levels of meaning from observations: what is explicit, what is implicit, and what is implied [63]. For example, a farmer’s observations of grazing lands turning into desert sands can involve the explicit sight of grass being replaced by sand. This explicit observation carries the implicit meaning that the farmer’s livestock cannot obtain necessary nutrition by grazing there. Thus, implicit observations of signals from the environment can relate to ecological fitness, i.e., the fit of the agent with the environment. Implicit observations of decreasing ecological fitness can carry the implication that it will not be possible to survive, for example, as a livestock farmer.

Pragmatic meaning can come from more than one observation at the same time. For example, a lorry driver can observe the sun setting behind a well-known landmark. Together, these explicit observations can carry the implicit meaning that there is not sufficient time remaining to travel the distance to the delivery destination before the deadline. This implicit meaning can carry the implication that the lorry driver’s employment will be terminated. It can carry this implication if the lorry driver has missed delivery deadlines in the past and has been threatened with unemployment if late again.

As the rational, the emotional, and the instinctual can be tangled together in human behavior [68], negative observations can be followed by what can appear to be irrational continuation or escalation of the same actions [69,70]. For example, when agents, such as livestock farmers and lorry drivers, have invested much in their current occupations and do not have any alternative occupations available to them, they can be more likely to ignore what is implicit and what is implied in explicit observations, and they keep trying to survive by persisting with current policies. This could involve, for example, looking farther and farther for grazing land and driving a lorry longer and faster without taking rest stops.

Observations can involve competing signals. For example, lorry drivers would rather not drive along a dangerous unlit road during the night. However, they may feel impelled to do so if observation of time signals indicates that the only hope of arriving by the delivery deadline, and so staying employed, is to drive through the night. Time signals are observed, for example, by seeing the sun setting, looking at a watch, etc. Yet, while driving through the night, the lorry driver can observe features of the environment that signal that it is not safe to continue, such as wrecks of lorries that have crashed in the past and small shrines for people who died on the road.

Amidst competing signals from observations, agents can engage in active inference. This involves three types of actions to bring about agents’ prior expectations for observations. In particular, observations that are consistent with their preferred states. The three types of actions in active inference are updating beliefs, shifting attention, and/or changing work method. For example, the lorry driver may update beliefs about the dangers of driving at night when a full moon in a cloudless sky illuminates the road. At the same time, the lorry driver needs to shift attention away from feelings of fatigue that have accumulated from already having driven for many hours without rest. This can be done by changing the method of work to include asking local villagers to run ahead of the lorry and use gestures to signal the depth of road cracks. Thus, the lorry driver can pay attention to external gestures rather than internal fatigue. This example illustrates that preferred states can be very far from ideal states.

Indeed, as shown by global shortages of lorry drivers, working in heavy haulage can be less than ideal even in rich countries [71,72] and can be inherently dangerous in other parts of the world [73,74]. Hence, lorry drivers’ prior expectations for observations that are consistent with preferred states are observations that are consistent with the preferred state of surviving. These can include many prior expectations for observations that lorry drivers would rather not experience, such as having to drive through day and night along dangerous roads. However, lorry drivers may do this if it is their only means of survival. In terms of competing signals, observations of many negative signals can be pooled together. For example, observations of many cracks in a road may be indistinguishable from each other as individually minor threats to survival. By contrast, threats of not being able to retain employment as a lorry driver can be separate from other observations that are pooled together [75,76]. Similarly, a livestock farmer can observe competing signals, one of which is separated from a pool of other signals. For example, there can be many observations of external signals arising from the sparsity of grazing lands that can be pooled together. By contrast, the internal signal of not believing that there is any other way of surviving than being a livestock farmer can be separate.

### 4.2. Concentration Parameters-Commitment Processes

Commitment to a course of action (i.e., to a policy) is influenced by the level of satisfaction with the policy, size of prior investment in the policy (i.e., sunk cost), and quality of alternative policies [65]. It has been argued that commitment processes exist in non-human as well as human life [77]. Notably, there can be increasing commitment to a failing policy even when this commitment can lead to extinction [78]. Commitment to a failing policy can involve risky behavior and motivated ignorance. In particular, if the magnitude of the expected reward is sufficiently high from a policy, agents will seek out the reward in the absence of sufficient information. For example, if the reward for a farmer is carrying on with the way of life that is all that the farmer and the farmer’s family have ever known, the farmer may display what could be described as the risky behavior of persisting with a failing course of action that threatens survival. This can involve the motivated ignorance of people choosing to avoid the costs that may come with obtaining new information about alternative policies [79]. For example, the physical disorder to livestock farmers of desertification is accompanied by information uncertainty about tasks and about identity. There is task ambiguity, for example, about where sufficient grazing land will be found today. There is identity risk, for example, about what will become of a committed livestock farmer in the future if desertification makes livestock farming impossible.

Thus, there can be continued strong commitment to a policy when satisfaction with the policy is low (e.g., trying to farm amidst desertification), but the size of sunk cost in the policy is high (e.g., generations of farming), and the quality of alternative policies is perceived as being low (e.g., abandoning an established way of life for identity uncertainty). Hence, people can continue to pay more attention to their out-of-date beliefs than to current sensory inputs. At the same time, people can avoid sensory inputs that contradict their out-of-date beliefs, such as not looking where the widest expanses of desertification can be seen, and they can approach sensory inputs that confirm their out-of-date beliefs, such as looking intensively for any indications of new grass growth.

Several methods can support analysis of commitment processes in practice. The size of prior investment in a current policy can be considered in terms of its path dependency. In particular, historical paths provide prior beliefs that influence the extent of learning from new sensory inputs. When more attention is paid to prior beliefs than to new sensory inputs, there will be inertia rather than learning [15]. Hence, addressing path dependencies involves recognition of, and reflection upon, historical paths of decisions that have led to current policies [80]. The quality of alternative policies can be considered in terms of the procedural, financial, relational, and collective costs of switching to them [81]. Analysis of resistance to changing from an existing policy to an alternative policy can be considered in terms of forces against change and forces for change within life spaces [82].

### 4.3. Actions–Psychomotor Functioning and Technological Mediation

Explicit observation of signals from the environment may or may not be consistent with the subsistence farmers’ predictions of what they will experience based on their generative models. There is low surprisal when what happens is what farmers have predicted will happen as a consequence of their actions. There is low surprisal because there are small information gaps between the farmers’ generative models and the generative process of the environment. Observations that are encountered frequently will have low surprisal, while observations that are encountered rarely will have high surprisal. Minimization of surprisal can be achieved through acting on the world and/or changing self [15].

For example, if the environment goes from supporting subsistence farming to fragmentation to collapse [83], farmers can act on the world and change themselves. Acting on the world can include expending more energy on actions such as searching for potential water sources and digging much deeper wells in more places after established water sources have dried out [84]. Thus, physical disorder and information uncertainty from environment fragmentation can lead to the entropy of energy not being available to perform productive work, such as caring for livestock that are stressed by water shortages [85].

Humans have long developed and applied technologies in order to mediate between such environmental challenges and survival preferences. Yet, there is a long ongoing pattern of human technologies addressing one environmental challenge but introducing other environmental challenges that are more difficult to address. For example, the development and use of water pumps has increased access to water in arid regions. However, pump technology depletes groundwater reserves and, while doing so, well yields decrease, pumping costs increase, water quality deteriorates, aquatic ecosystems are damaged, and land subsides irreversibly. Nonetheless, despite the depletion of groundwater reserves having been observed for decades, pump technology continues to be deployed more widely [86,87].

At the same time, technologies that are developed and used to make human work less strenuous can have negative unintended consequences for people’s psychomotor functioning [88]. Thus, technologies that are developed with good intentions have led to negative consequences for people acting on the world and for people changing themselves. Negative effects can be on complex mind–body behaviors, which include interactions between, for example, personality type and body memory [67]. Changes to self can include suffering from multiple interrelated mental and physical health issues [89]. Hence, although commitment to continuing with a failing course of action can override negative observations, such commitment is not sufficient to prevent psychomotor functioning being undermined. Thus, commitment to a failing course of action cannot carry on indefinitely as psychomotor functioning is necessary for survival.

### 4.4. Synthesis

Table 2 provides a summary of unsustainable interactions between observations, policy commitment, and actions. In Table 2, these are actions when an environment goes from supporting subsistence farming to fragmentation to collapse [83]. Notably, there are no better policy options as implications for survival become increasingly negative. As discussed above, policy commitment is influenced by satisfaction with the policy, sunk cost in the policy (i.e., size of investment in the policy), and the quality of alternative policies. For brevity, the quality of alternative policies is summarized in Table 2 as whether or not a better policy is available. As the environment deteriorates, policy satisfaction decreases and sunk cost increases as more investment has to be made to survive. For example, more and deeper wells have to be dug to obtain water. At the same time, there is no better policy available to the subsistence farmers. Thus, there is no competing signal to those observed by the farmers as they try to maintain their long-established subsistence way of life. In particular, there is no competing signal for explicit signals such as more and more sand replacing an increasingly wide region of what used to be grasslands. This explicit observation carries the implicit meaning of reduced fit with the environment, which carries the implication that it will not be possible to survive as a livestock farmer.

Overall, the summary in Table 2 shows that environment deterioration brings negative effects for surprisal, fitness, and survival as technological mediation and psychomotor functioning are not able to address environmental deterioration. By contrast, Table 3 provides a summary of regenerative interactions between observations, policy commitment, and actions. This shows that environmental fragmentation is addressed because there is a better policy option and alternative technology available compared to carrying on with the previous policy option. This is because there has been increasing recognition of the need to improve how technologies mediate between people and the environment. In terms of ecosystem engineering, where people make widespread environmental changes across countries and continents, agroecology is an example of technology mediation to bring about positive global balance between human production and environmental systems [90]. In terms of niche construction, where people change a local environment, the Songhai Center at Porto Novo in Benin is an example founded in agroecology that extends to the entrepreneurial local development and use of agricultural machinery by local people. Songhai has stated commitment to bioenergy, bio-production, bioprocessing, and bio-consumption. Bio-production involves protecting the environment’s natural resources by integrating growing agricultural crops, raising fish, livestock husbandry, and producing energy. Bioprocessing involves agrifood products being made from local organically grown products that are processed locally. Bio-consumption involves providing local markets, restaurants, and hotels. Overall, Songhai processes are intended to manage flows of bioenergy and biomass in order to generate new and better biological capital, while producing food in sufficient quantities to promote human health and generate new agroecology enterprises that pay attention to signals from the environment [91,92,93]. Thus, Songhai provides an example of synchronous generative development that balances the well-being of people and the environment. In doing so, there is a positive balance across situated entropy experienced by people and by the environment. For both, agroecological technology mediation brings some changes compared to the physical order that emerges through many millennia in nature. These changes bring some physical disorder and some information uncertainty, but these are within the scope of positive balance in human and in environmental generative development. There is no attempt to suppress human and environmental diversity in order to bring situated entropy to zero across all actions. For example, there is no attempt to impose a continuous monoculture. Rather, individual self-expression is encouraged. For example, up to 70 percent of graduates from Songhai Center succeed in engaging in their own agribusiness activities [94]. Here, it is important to note that a positive balance of situated entropy does not equal zero situated entropy for both people and for the environment. For example, brain entropy, which is the number of neural states a given brain can access, is associated with intelligence [95] and with creativity [96]. More broadly, diversity in nature fosters open-ended evolution of ecological fitness [97,98]. Thus, a positive balance of situated entropy between people and environment is a balance that reduces physical disorder and information uncertainty but does not focus on entirely eliminating either. In other words, there is a balance between entropy pessimism [26] and entropy optimism [99], within which reducing entropy can increase efficiency but not to such an extent that there is no flexibility to develop amidst disturbances [7,8].

## 5. Federated Digital Twins for Synchronous Generative Development

### 5.1. Federated Digital Twins

Digital twins are digital models that mirror physical processes; that are connected to physical processes through, for example, sensors and actuators; and which carry out analyses of physical processes in order to improve their performance. Federated digital twins comprise several different digital twins that share data [16,17]. The physical process can be conceptualized as the generative process and the digital model can be conceptualized as the generative model. Sensors enable observations and actuators enable action. Thus, digital twins are consistent with key constructs in synchronous generative agent-environment systems. In addition, both digital twins and synchronous generative agent-environment systems [14,15] are consistent with the conceptualization that every good regulator of a system must be a good model of that system [100]. A good model is a model with a structure that mirrors the most ecologically relevant aspects of the environment [101]. Furthermore, within the physics of life framework that underlies synchronous generative agent-environment systems [14,15], it is postulated that the brain embodies an internal model of the world that is generative in the sense that it can simulate the sensory data that it should receive if its model of the world is correct. These simulated (i.e., predicted) sensory data can be compared to actual observations, and differences between predicted and observed sensations can be used to update the model [42]. Similarly, analyses carried out by digital twins often involve model-based simulations [43]. Moreover, key constructs in synchronous generative agent-environment systems’ generative models are important for digital twin simulation models. In particular, there is a trade-off between accuracy and complexity, within which the model should be as accurate as possible, but complexity should be minimized in order to facilitate reliable economic updating. The model should also be focused on minimizing risk to the operation of the physical process and on minimizing the ambiguity of data collected from the physical process. Thus, digital twins have important similarities with synchronous generative agent-environment systems.

Digital twins can be applied to urban agroecological processes [102]. For example, digital twins are applicable to soil-less agriculture, such as hydroponics and aquaponics. Hydroponics is a soil-less growing system where roots of plants are submerged in the nutrient solution. Aquaponics combines aquaculture and hydroponics by feeding aquatic animals discharge into hydroponics tanks [103,104]. Digital twins can also be applied to vertical farming where food is grown on the walls and on the roofs of buildings with controlled irrigation for minimal water consumption [105]. More broadly, there has been consideration of potential to apply digital twins to urban agroecological methods in urban agriculture [106]. A widespread example of urban agroecology is urban allotments [107] to which digital twins for so called smart farming are applicable [108]. Different means of observations and actions are involved in different urban agricultural processes. For example, aquaponics in controlled internal environments can involve extensive observations and actions with automated sensors and actuators. Similarly, automated sensors and actuators can facilitate vertical farming on the outside of high-rise buildings. Actuators can carry out actions such as opening and closing water valves. However, harvesting actions can be difficult to automate. Importantly, citizens can be involved in the participatory design of technology deployments that involve automated data collection with sensors [109]. Some sensing can also be reliant on human observations in so called participatory sensing [110], for example, the observations of which fruits and vegetables are ripe at an urban allotment. More broadly, it is possible to facilitate participatory sensing through so-called citizen observatories. These build on citizen science methodologies to engage citizens in community-based environmental monitoring. For example, citizens can be trained as citizen scientists who collect and analyze data using a variety of techniques and technologies [111]. Notably, it has been argued that citizen science can contribute to widening agroecology implementation and to realization of SDGs [112,113].

Overall, the development of digital twin deployments should be a work of collective imagination involving participants from across communities [114], which takes into account different perceptions and norms [115]. Such participatory design can take into account established design principles for community-based natural resource management [116]. In Table 4, digital twins are related to constructs in synchronous generative agent-environment systems through the example of urban agriculture. Key constructs are generative process, generative model, observations, commitment, actions, accuracy, complexity, risk, ambiguity, and synchrony.

While sensing for aquaponics and vertical farming, digital twins can be fully automated. By contrast, sensing for urban allotments can rely on human observations that are communicated through, for example, simple text message options. Focus of commitment can be different for different urban agricultural options. Private sector aquaponics entrepreneurs’ commitment can be founded upon a large financial sunk cost, whereas individuals who tend their own allotments can have a large sunk cost of time and effort. Accordingly, risks can be different but still related to survival. Risk can be thought of as expected divergence between predicted and preferred outcomes. In other words, risk can be thought of as beliefs about the probability of reward for each choice one could make. Risk can be framed as being a hierarchy that begins with task risks, that can extend to identity risks, and to existential risk. Task risks for the aquaponics entrepreneur begin at the level of valves not regulating processes, extend to the identity risk of being a failed entrepreneur, and can lead to not being able to survive bankruptcy. Accordingly, it is imperative to minimize the ambiguity of sensory inputs at the level of task risks because if the aquaponics nutrient solution condition is not good, but sensors indicate that it is good, the aquaponics enterprise could fail. To minimize ambiguity, the digital twin setup must facilitate accurate observations and actions. Accuracy can be facilitated by managing complexity.

### 5.2. Situated Entropy

Situated entropy can have a determining influence over complexity. Physical disorder in aquaponics can be low due to being situated inside buildings. By contrast, physical disorder from vertical farming situated on the outside of tall buildings can be higher. Compared to these two options, urban allotments situated at ground level may experience medium physical disorder. Aquaponic systems are engineered to minimize physical disorder and information uncertainty to minimize unproductive energy consumption. Compared to an urban allotment, the engineering of aquaponics systems involves far more initial energy expenditure. However, aquaponics systems can be scaled out through modular kits [117]. Thus, the initial energy expenditure can be spread out over many installations that have low unproductive energy consumption in their operation. Also, vertical farming can be scaled out through modular kits. Physical disorder and information uncertainty in vertical farming can be reduced by situating inside buildings [118]. Furthermore, physical disorder, information uncertainty, and unproductive energy consumption of urban allotments can be reduced through initial energy expenditure in careful planning of, for example, plant types and irrigation methods [119].

Yet, the complexity of human-driven biosocial-technical systems can be increased by having simulation models in digital twins as well as generative models in human minds. For example, from the point of view of digital twins, information uncertainty may be highest for the urban allotments because incoming data depends upon human inputs rather than upon automated sensors. By contrast, from the point of view of people, information uncertainty may be highest for vertical farming because it is most difficult to be able to see the condition of plants close up. Thus, a digital twin may analyze a vertical farm to be in bad condition, but a human may view the same vertical farm as being in good condition. Furthermore, this may be because the human observations may be influenced by escalating commitment to a failing vertical farming installation. For example, people may consider that they will become unemployed (implied) if they are responsible for large public sector investments in failed vertical farming (implicit) as would be signaled by a bad condition such as plants being brown instead of green (explicit). At the same time, a subcontractor providing digital twin services may be reluctant to question the view of the public sector officials who are responsible for authorizing the payments needed for the subcontractor to survive. Hence, there is potential for information uncertainty between a digital model developed by humans and the different humans’ own mental models. This can lead to physical disorder and unproductive energy consumption as humans try to take a closer look at the plants in the vertical farm installation and eventually take legal proceedings against each other in order to allocate financial responsibility for the failure of a vertical farm installation.

Potential for situated entropy can increase if the federation of digital twins is complex. Accordingly, the structuring of digital twins and their interconnection for data exchange should be sparse and should be transparent [120]. Nonetheless, more than technical engineering is required for minimizing unproductive energy expenditure [121]; there needs to be recognition from the outset of potential for physical disorder and information uncertainty arising from conflict among human mental models. For example, agroecology technologies can contribute to mediating between scarce water resources and human demand for water. Yet, decisions about the supply of piped water to urban agroecological farming can be affected by competing demand from a wide range of people with different mental models. Such competition can involve digital twins that encompass a very wide range of activities in the same city [122]. However, digital twins do not encompass the social power of different parties competing for water supply. For example, one city authority that has invested heavily in vertical farming can have more influence over the allocation of scarce water resources than a few aquaponics entrepreneurs and many allotment farmers. As water supply is essential to psychomotor functioning, it can be anticipated that all people will have a fundamental commitment to opposing any threat to water supply. A summary of situated entropy variables is provided in Table 5. This highlights that the entropy of energy being unavailable for productive work can depend upon the amount of unproductive energy expenditure arising from potential for physical disorder, for example, from the microclimate of high-rise building with a vertical farming installation. Furthermore, there can be unproductive energy consumption if a human does not agree with the analyses of digital twins and does not agree with other people about the allocation of piped water supplies. Thus, even though agroecology is compatible with synchronous generative agent-environment systems, there can be conflict between different human priorities and different digital models, and that can lead to unproductive energy expenditure. Accordingly, it is important that potential conflicts between different participants’ priorities are addressed in the participative design of urban agroecology, digital twins, and interactions between them. Agroecology’s tripartite foundation, in scientific discipline, social movement, and sustainable practice [20] can provide examples into how potential conflicts can be addressed [123,124].

## 6. Conclusions

### 6.1. Principal Contributions

Joint agent-environment systems theory is focused on reciprocal synchronous generative development. The purpose of this paper is to extend this theory towards practical application in sustainable development projects, in particular, those involving digital twins. In Figure 1, Figure 2 and Figure 3, description for practitioners employs the radar chart format that is applied widely in organizational practice. As summarized in Table 2 and Table 3, theory extension is also made through synthesis with research concerned with multilevel pragmatics, competing signals, commitment processes, technological mediation, and psychomotor functioning. In addition, the extended theory is related to federated digital twins for agroecological urban farming. As summarized in Table 4 and Table 5, this example indicates the complexity that can arise in human-driven biosocial-technical systems, even when individual implementations of agroecology are compatible with synchronous generative development.

A limitation of the paper is that the examples provided in the paper encompass only few of the many types of agents that can interact with each other in environments. Nonetheless, the formulation of situated entropy in this paper is relevant to many different agents in many different environments. For example, a person driving an ox cart can experience high situated entropy in a typical urban setting when there is low synchrony between the agent (i.e., the ox) and the environment (i.e., hard paved urban landscapes). By contrast, a person driving an ox cart can experience low situated entropy when in high synchrony with an urban agroecological setting (i.e., soil-based urban landscape). Thus, interrelationships between synchrony and situated entropy should be an important consideration in the development of so called post-anthropocentric cities, within which humans and non-humans are interdependent [125]. Another limitation of the paper is that it does not encompass creativity in generative models. Nonetheless, a generative model can involve the prediction of future environments. In particular, people can imagine themselves within future environments and consider simultaneously what would happen if we did that, what would we think about it, and what would other people think about it [126,127,128]. Accordingly, generative models are relevant to participatory design of public spaces for urban agriculture [129].

### 6.2. Practical Implications

Agent-environment systems theory can provide a unifying framework for physical actions, digital twins of physical actions, and interactions between them in development projects. Importantly, joint agent-environment systems theory frames sustainable development as being dependent on only one goal, which is reciprocal synchronous generative development. Focusing on this one overarching goal can provide a more straightforward starting point for the development projects than trying to align with 17 Sustainable Development Goals that are, to some extent, in conflict with each other. In addition, key constructs in agent-environment systems’ theory are applicable to the formulation and operation of digital twins. Those key constructs are complexity, accuracy, risk, and ambiguity. In particular, digital twins’ complexity and their predictive accuracy should be balanced during the formulation of digital twins. During their operation, digital twins should be focused on minimizing risk to physical processes and on minimizing the ambiguity of data collected from the physical processes. Furthermore, agent-environment systems theory and its underlying physics of life principles bring together information-theoretic entropy and thermodynamic entropy, both of which are applicable to physical actions and digital twins of physical actions. Thus, agent-environment systems theory can provide a starting point for addressing situated entropy in physical actions, digital twins, and interactions between them.

### 6.3. Future Research

As summarized in Table 4 and Table 5, complexity can arise from human-driven biosocial-technical systems, such as digital twins, even when they are compatible with synchronous generative development. Accordingly, future research should focus on addressing this potential for complexity that can undermine efforts to minimize risk. Such research should recognize from the outset that digital twins have the potential to introduce new sources of situated entropy. For example, their functioning can be affected negatively by the vulnerability of sensors and actuators to physical disorder that brings information uncertainty that can lead to unproductive energy expenditure. Moreover, digital twins can provide analysis results that are accurate, but which are in conflict with human generative models that are governed by commitment to course of action irrespective of sensory inputs. It is important to address these sources of complexity because they can inadvertently lead to situated entropy that reduces the energy available for synchronous generative development. Accordingly, an interesting direction for future research is to investigate the practicality of designing digital twins in accordance with the human ability to generate accurate inferences from very few sensory inputs [130]. Such research can draw upon the latest findings from neuroscience and from machine learning, both of which indicate that simple models have the potential to be as accurate as complex models [42,118]. An example of this is model reduction in which redundant model parameters are removed to prevent model over-fitting and promote selection of the most parsimonious model that can successfully account for previous observations [42]. A related direction for further research is participative design of digital twins. For example, consideration could be given to the use of multimodal symbol systems to facilitate the development of mutual knowledge among participants who have different backgrounds and languages that can introduce information-theoretic entropy into the design process [131].

## Figures and Tables

**Table 1:** Constructs.

Construct	Description
Generative process	Generates agents’ observations of the world.
Generative model	Generates agents’ expectations about the world.
Observations	Observed sensory inputs coming from the generative process.
Commitment	Commitment to a course of action influences how much attention agents pay to observations.
Actions	Courses of action are influenced by observations and commitment.
Accuracy	Generative model should be as accurate as possible in its expectations about observations.
Complexity	Complexity of generative model should be minimized to facilitate its reliable economic updating.
Risk	Generative model should be focused on minimizing risk to survival.
Ambiguity	Generative model should minimize ambiguity of observations from the generative process.
Synchrony	Reciprocal exchanges of learning and development between agent and environment.

**Table 2:** Unsustainable interactions between observations, policy commitment, and actions.

Environment	Agents							
	Observations			Policy Commitment			Actions	
	Explicit Surprisal	Implicit Fitness	Implied Survival	Satisfaction	Sunk Cost	Better Option	Technology Mediation	Psychomotor Functioning
Subsistence	Low	High	Yes	High	Low	No	High	High
Fragmented	Medium	Medium	Maybe	Medium	Medium	No	Medium	Medium
Collapsed	High	Low	No	Low	High	No	Low	Low

**Table 3:** Regenerative interactions between observations, policy commitment, and actions.

Environment	Agents							
	Observations			Policy Commitment			Actions	
	Explicit Surprisal	Implicit Fitness	Implied Survival	Satisfaction	Sunk Cost	Better Option	Technology Mediation	Psychomotor Functioning
Subsistence	Low	High	Yes	High	Low	No	High	High
Fragmented	Medium	Medium	Maybe	Medium	Medium	Yes	Medium	Medium
Regenerated	Low	High	Yes	High	Medium	No	High	High

**Table 4:** Synchronous generative agent-environment systems related to digital twins.

Construct	Example		
	Aquaponics	Vertical Farming	Urban Allotments
Generative process	Feeding of aquatic animals’ discharge into hydroponics	Plants growing on sides and roofs of buildings	Plants growing in soil at small plots of land
Generative model	Digital twin of aquaponics tanks input/output valves	Digital twins of vertical farming irrigation valves	Digital twin of allotment soil conditions
Observations	Automated sensors	Automated sensors	Human observations sent to digital twin via text messages
Commitment	Private sector financial investment in equipment	Public sector financial investment in equipment	Personal investment of time and effort
Actions	Automated valves	Automated valves	Manual tending of soil/plants
Accuracy	Valve operation to maintain the best nutrient solution	Valve operation to maintain best irrigation levels	Human assessment and tending of soil/plant
Complexity	Valves’ number, variety, and operating parameters	Valves’ number, variety, and operating parameters	Human behavior in tending of soil and plants
Risk	Failed private investment	Failed public investment	Insufficient food to survive
Ambiguity	Nutrient solution condition	Plant hydration levels	Condition of soil and plants
Synchrony	Synchronization between physical processes, digital models, and human mental models		

**Table 5:** Situated entropy related to synchronous generative agent-environment system of urban agroecology.

Situated Entropy		Examples of Relative Potential for Situated Entropy		
		Aquaponics	Vertical Farming	Urban Allotments
Physical Disorder	Automation	Low	High	Medium
	Human	Low	High	Medium
Information Uncertainty	Automation	Low	Medium	High
	Human	Medium	High	Medium
Unproductive energy expenditure	Automation	Low	Low	Medium
	Human	Depends on agreement with digital twin and with people competing for water supply	Depends on extent of physical disorder at heights, on agreement with digital twin, and with people competing for water supply	Depends on extent of physical disorder on the ground, on agreement with digital twin, and with people competing for water supply

## Data Availability

Not applicable.
